# Supplementary material for: Co-evolution of matrisome and adaptive adhesion dynamics drives ovarian cancer chemoresistance
Source: Nat Commun. 2021 Jun 23;12:3904. doi: 10.1038/s41467-021-24009-8 (PMC8222388; doi:10.1038/s41467-021-24009-8)
Supplement: Supplementary file 1 — Supplementary Information [file 41467_2021_24009_MOESM1_ESM.pdf]

Supplementary Information

Pietilä et al.,
Co-evolution of matrisome and adaptive adhesion dynamics drives ovarian cancer chemoresistance

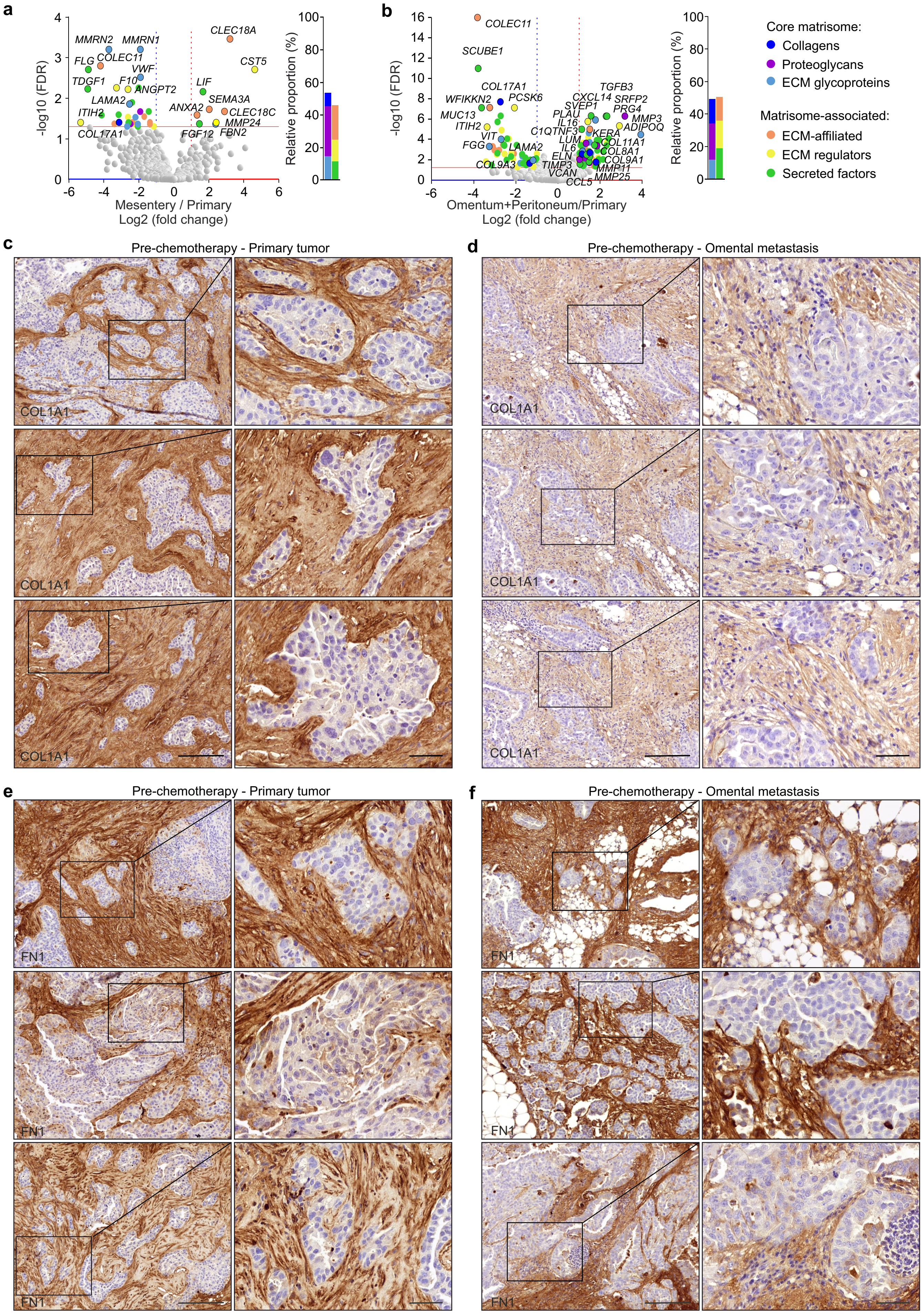
a
-log10 (FDR)
MMRN2
MMRN1
CLEC18A
FLG
COLEC11
CST5
VWF
TDGF1
F10
LIF
ANGPT2
LAMA2
SEMA3A
ANXA2
ITIH2
CLEC18C
MMP24
COL17A1
FGF12
FBN2
Mesentery / Primary
Log2 (fold change)
Relative proportion (%)
b
COLEC11
SCUBE1
COL17A1
TGFB3
WFIKKN2
PCSK6
CXCL14
SRFP2
SVEP1
PRG4
MUC13
PLAU
MMP3
ITIH2
IL16
ADIPOQ
C1QTNF3
VIT
KERA
LUM
COL11A1
FGG
LAMA2
IL6
COL8A1
ELN
COL9A1
COL9A3
TIMP3
MMP11
VCAN
MMP25
CCL5
Omentum+Peritoneum/Primary
Log2 (fold change)
Relative proportion (%)
Core matrisome:
Collagens
Proteoglycans
ECM glycoproteins
Matrisome-associated:
ECM-affiliated
ECM regulators
Secreted factors
c
Pre-chemotherapy - Primary tumor
COL1A1
COL1A1
COL1A1
d
Pre-chemotherapy - Omental metastasis
COL1A1
COL1A1
COL1A1
e
Pre-chemotherapy - Primary tumor
FN1
FN1
FN1
f
Pre-chemotherapy - Omental metastasis
FN1
FN1
FN1

Supplementary Figure 1. HGSC cell colonies are enclosed by dense fibrotic stroma in primary tumor and metastatic tissue sites.
a,b, Volcano plots and corresponding charts depict differentially expressed genes (DEGs) encoding matrisome proteins in mesenteric metastasis (**a**; n = 6) and in combined omental and peritoneal metastases (**b**; n = 49) against primary tumors (n = 32). In volcano plots, colored dots indicate DEGs of each matrisome category: dark blue = collagens, purple = proteoglycans, light blue = extracellular matrix (ECM) glycoproteins, orange = ECM-affiliated proteins, yellow = ECM regulators, green = secreted factors. Horizontal line shows Benjamin-Hochberg-adjusted p-value < 0.05 (FDR, false discovery rate); vertical lines depict 2.0-fold increased (red) and decreased (blue) expression. Bars in charts depict the relative proportion of DEGs within the 6 different matrisome categories.
c-f, Micrographs of collagen 1A1 (COL1A1, **c-d**) and fibronectin (FN1, e-f) immunohistochemistry of pre-chemo primary tumor (n = 2) and omental metastasis (n = 6). Scale bar = 200 μm and 50 μm in insets.

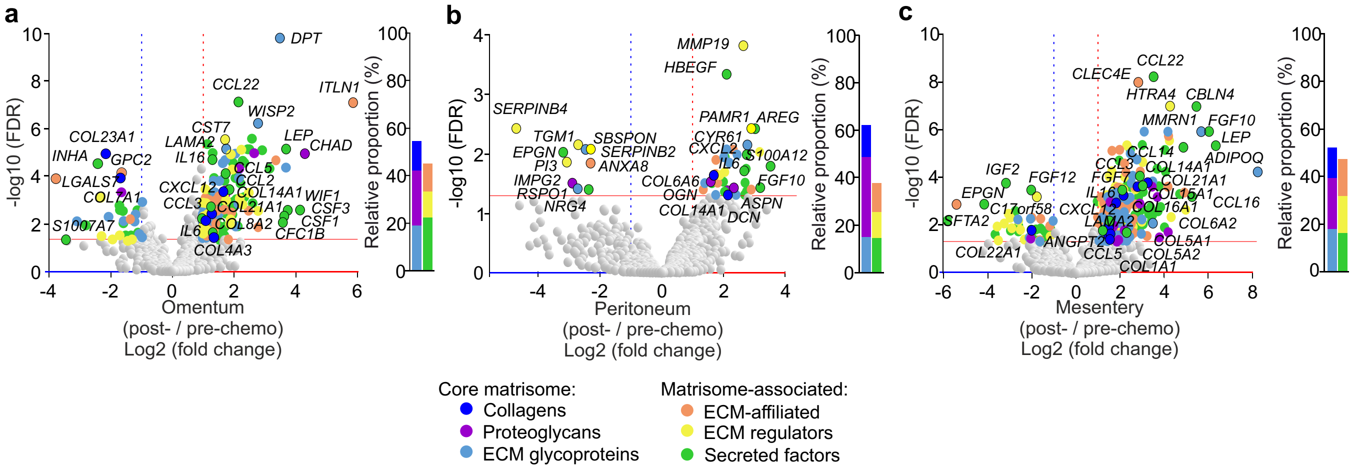

Supplementary Figure 2. Core ECM proteins are upregulated in solid HGSC tumors upon chemotherapy.
a-c, Volcano plots and corresponding charts depict differentially expressed genes (DEGs) encoding matrisome proteins in post-chemotherapy omental (**a**; n = 23 vs 21), peritoneal (**b**; n = 5 vs 28) and mesenteric (**c**; n = 10 vs 6) metastases compared to matching pre-chemotherapy samples. In volcano plots, colored dots indicate DEGs of each matrisome category: dark blue = collagens, purple = proteoglycans, light blue = extracellular matrix (ECM) glycoproteins, orange = ECM-affiliated proteins, yellow = ECM regulators, green = secreted factors.
Horizontal line shows Benjamin-Hochberg-adjusted p-value < 0.05 (FDR, false discovery rate); vertical lines depict 2.0-fold increased (red) and decreased (blue) expression. Bars in charts depict the relative proportion of DEGs within the 6 different matrisome categories.

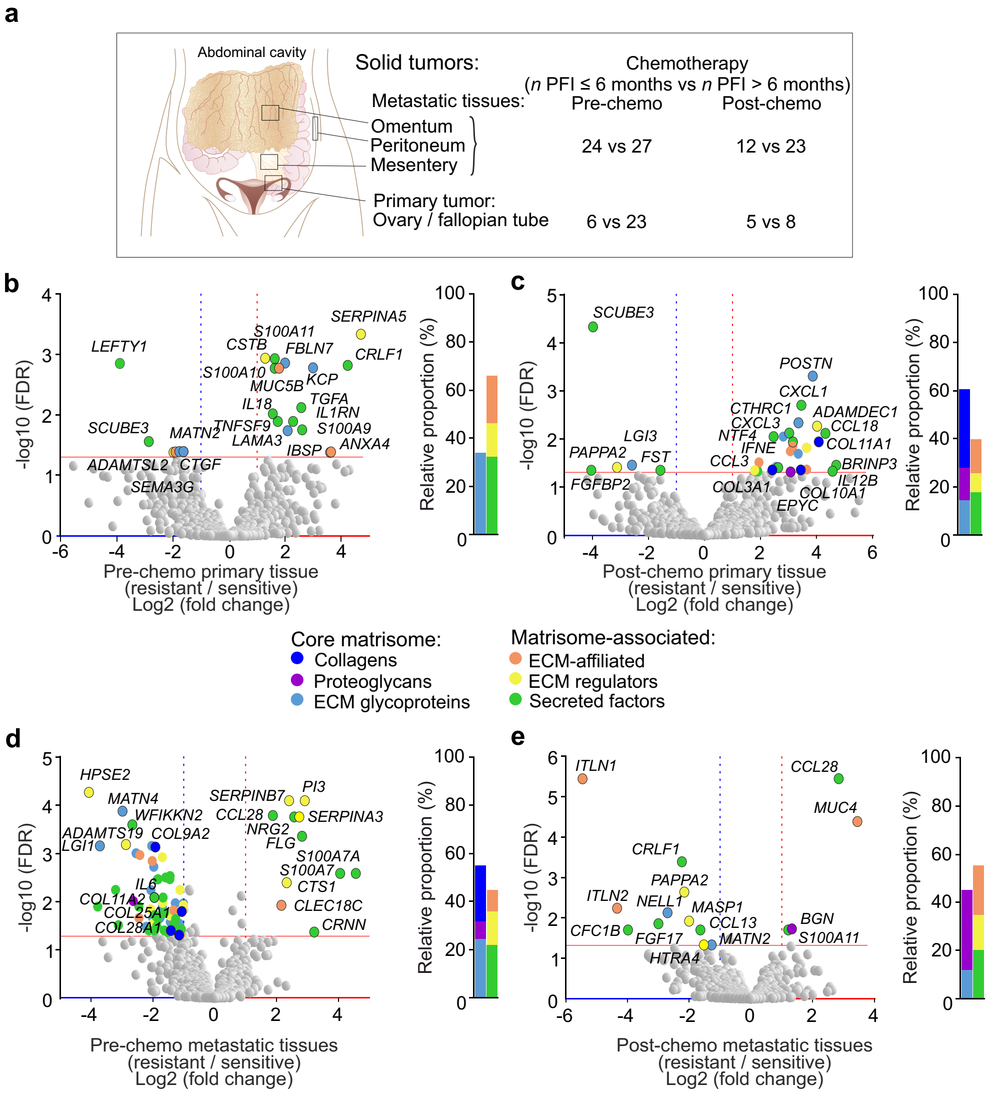

Supplementary Figure 3. DEGs in patients with platinum sensitive versus platinum resistant disease.

a, Schematic diagram showing the anatomical locations and the types of samples collected from high grade serous carcinoma (HGSC) patients with platinum resistant (platinum-free interval/PFI) ≤ 6 months) or platinum sensitive (PFI > 6 months) disease. n = number of samples.

b-e, Volcano plots and corresponding charts depict differentially expressed genes (DEGs) encoding matrisome proteins in pre-chemo primary tissue (b; n = 6 vs 23), post-chemo primary tissue (c; n = 5 vs 8), pre-chemo combined metastases (d; n = 24 vs 27), and post-chemo combined metastases (e; n = 12 vs 23) from platinum resistant against platinum sensitive patients. In volcano plots, colored dots indicate DEGs of each matrisome category: dark blue = collagens, purple = proteoglycans, light blue = extracellular matrix (ECM) glycoproteins, orange = ECM-affiliated proteins, yellow = ECM regulators, green = secreted factors.

Horizontal line shows Benjamin-Hochberg-adjusted p-value < 0.05 (FDR, false discovery rate); vertical lines depict 2.0-fold increased (red) and decreased (blue) expression. Bars in charts depict the relative proportion of DEGs within the 6 different matrisome categories. See Supplementary Data 24, 25 for DEGs in ascites-derived cancer cells.

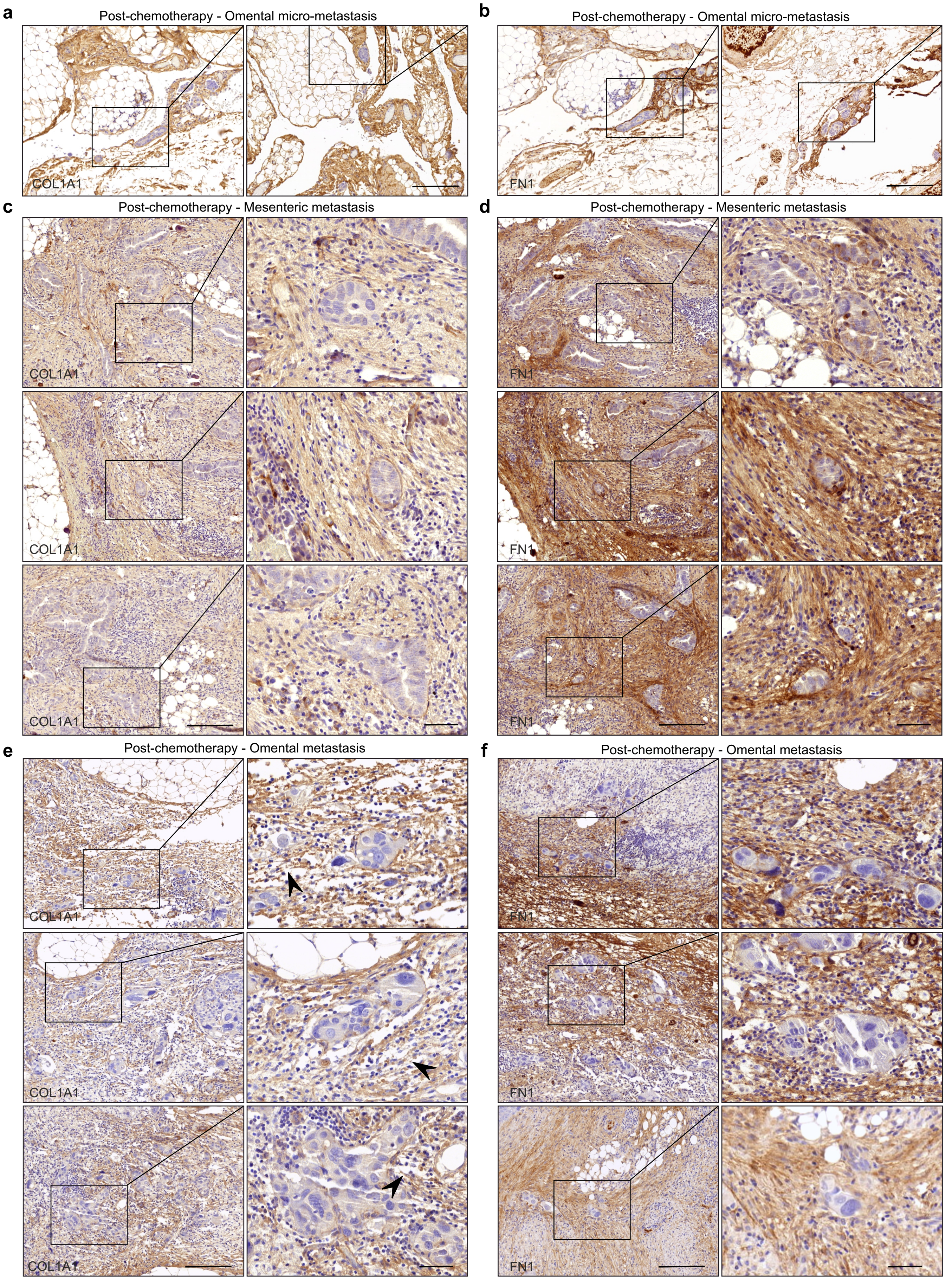

Supplementary Figure 4. ECM fibers are compromised in post-chemo mesenteric and omental metastases.
a,b, Lower magnification micrographs from insets in Fig. 2. Images representative of n = 2 patients. Scale bar = 200 µm.
c-f, Micrographs of collagen 1A1 (COL1A1) and fibronectin (FN1) immunohistochemistry of post-chemo mesenteric (**c, d,** n = 2) and omental metastasis (**e, f,** n = 10). Arrowheads indicate fragmented collagen fibers. Scale bar = 200 µm and 50 µm in insets.

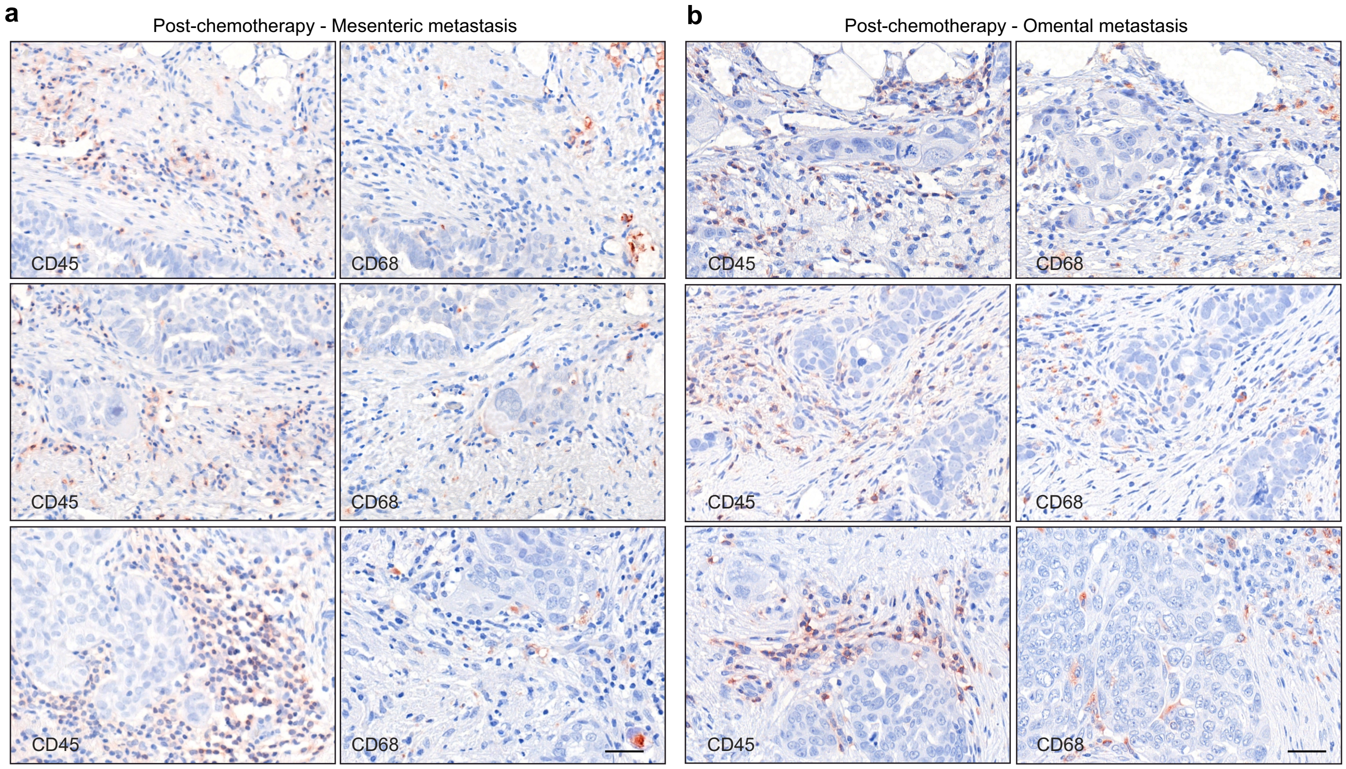

Supplementary Figure 5. Leukocytes in post-chemo mesenteric and omental metastases.
a,b, Micrographs of CD45 and CD68 immunohistochemistry of post-chemo mesenteric (n =1; **a**) and omental (n = 4; **b**) metastasis. Scale bar = 50 µm.

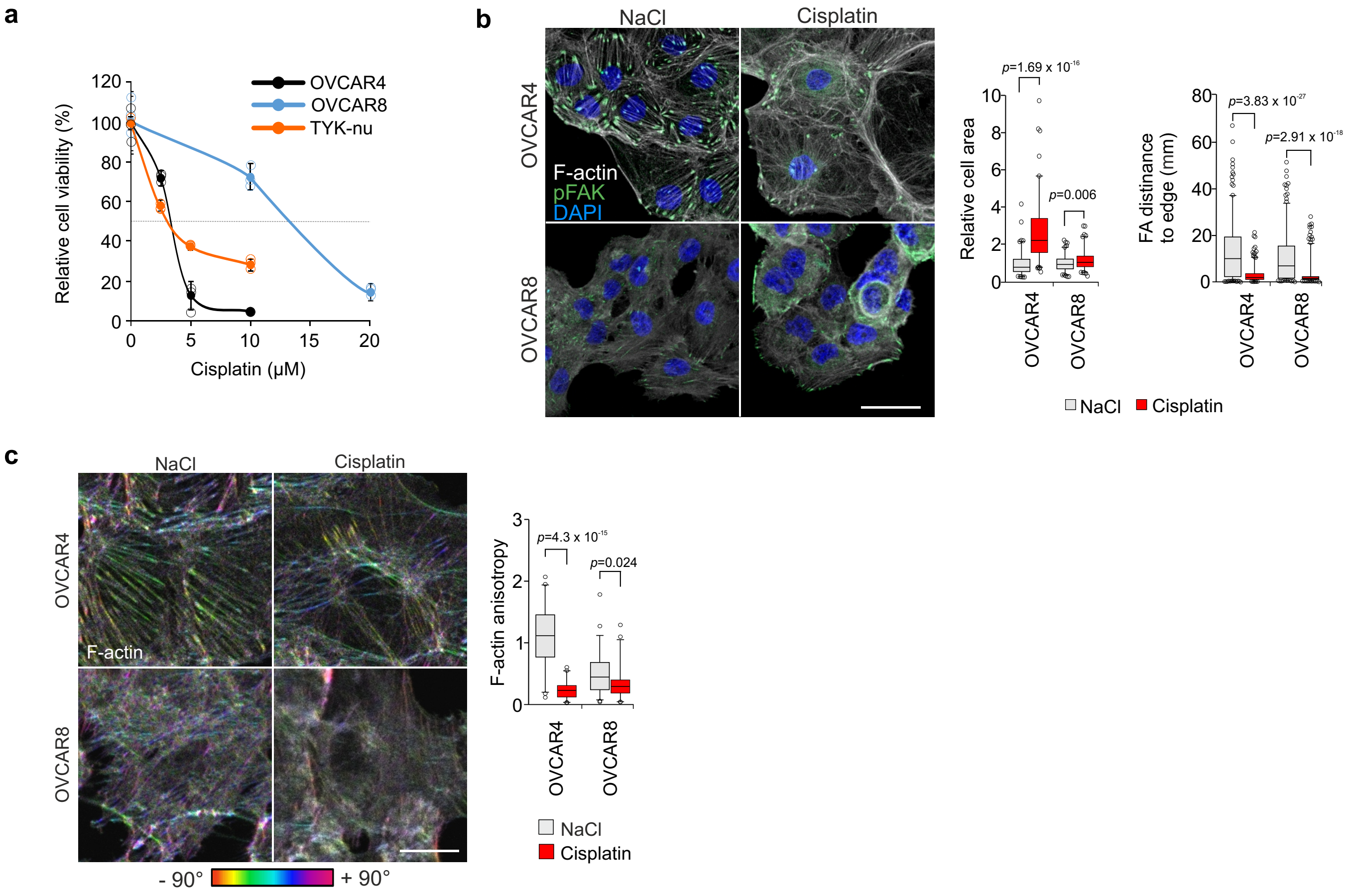

Supplementary Figure 6. Stiff matrix controls ECM-focal adhesion signaling.
a, Scatter plot illustrates cell viability after 72 h treatment with 0-20 µM cisplatin in OVCAR4, OVCAR8 and TYK-nu. Cell viability was determined by ATP measurement and is shown in reference to NaCl (control).
b, Representative confocal micrographs of F-actin (phalloidin, white) and phosphorylated focal adhesion (pFAK, green) in OVCAR4 and OVCAR8 grown on glass coverslip and treated with 2 µM (OVCAR4) or 10 µM (OVCAR8) cisplatin for 48 h. Charts illustrate the cisplatin-induced cell area (n = 100 cells), shown relative to NaCl treatment (control) per cell line, as well as peripheral localization of FAs (OVCAR4 n = 259/244 and OVCAR8 n = 234/256 cells, respectively). Scale bar = 25 µm.
c, Representative confocal micrographs of F-actin show the decreased F-actin anisotropy in OVCAR4 and OVCAR8 grown on glass coverslip and treated with 2 µM (OVCAR4) or 10 µM (OVCAR8) cisplatin for 48 h; OVCAR4 n = 44/39 and OVCAR8 n = 57/51 cells, respectively; the color key identifies the angular orientation of actin fibers. Scale bar = 10 µm.
Data represent mean ± SEM; n = 3 biological replicates; two-tailed Student's t-test. Box plots indicate median (middle line), 25th, 75th percentile (box) and 10th and 90th percentile (whiskers) as well as outliers (single points).
Source data are provided as a Source Data file.

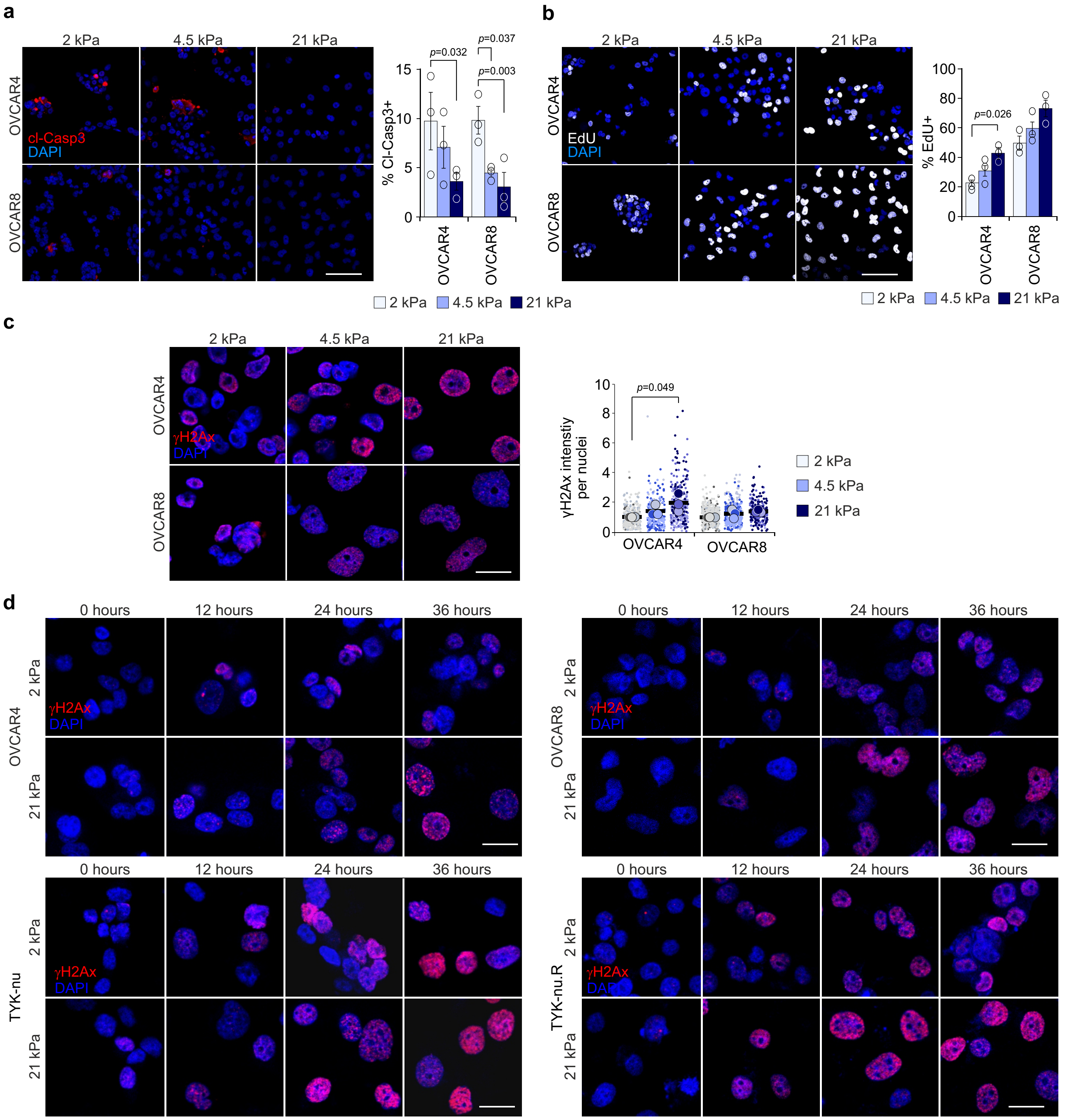

Supplementary Figure 7. Stiff ECM protects both cisplatin-sensitive and resistant HGSC cells against platinum-induced apoptosis.

a, Representative confocal micrographs and corresponding quantifications of cleaved caspase 3 (cl-Casp3, red; marker of apoptosis) in OVCAR4 and OVCAR8 grown on 2 kPa, 4.5 kPa and 21 kPa collagen 1 functionalized polyacrylamide (COL1-PAA) hydrogels and treated for 24 hrs with IC30 cisplatin: OVCAR4 2 μM and OVCAR8 10 μM, see Supplementary Fig. 6a. Scale bar = 50 μm.

b, Representative confocal micrographs and corresponding quantifications of EdU (white, marker of proliferation) in untreated OVCAR4 and OVCAR8 grown on 2, 4.5 and 21 kPa COL1-PAA hydrogels Scale bar = 50 μm.

c, Representative confocal micrographs and corresponding quantification of phosphorylated H2Ax (γH2Ax, red) in OVCAR4 and OVCAR8 on corresponding COL1-PAA. Cells were treated with 2 μM (OVCAR4) or 10 μM (OVCAR8) cisplatin for 24 h. Superplots depict each cell within the color-coded replicate and their mean (2 kPa, grey; 4.5 kPa, light blue; 21 kPa, dark blue). Scale bar = 10 μm.

d, Representative confocal images of γH2Ax (red) in corresponding cells on 2 and 21 kPa COL1-PAA and treated with 2 μM (OVCAR4, TYK-nu, TYK-nu.R) or 10 μM (OVCAR8) cisplatin up to 36 h. Scale bar = 10 μm. See Fig. 3e for quantifications.
Data represent mean ± SEM; n = 3 (**a**,**c**) and n = 4 (**b**) biological replicates; two-tailed Student's t-test. Source data are provided as a Source data file.

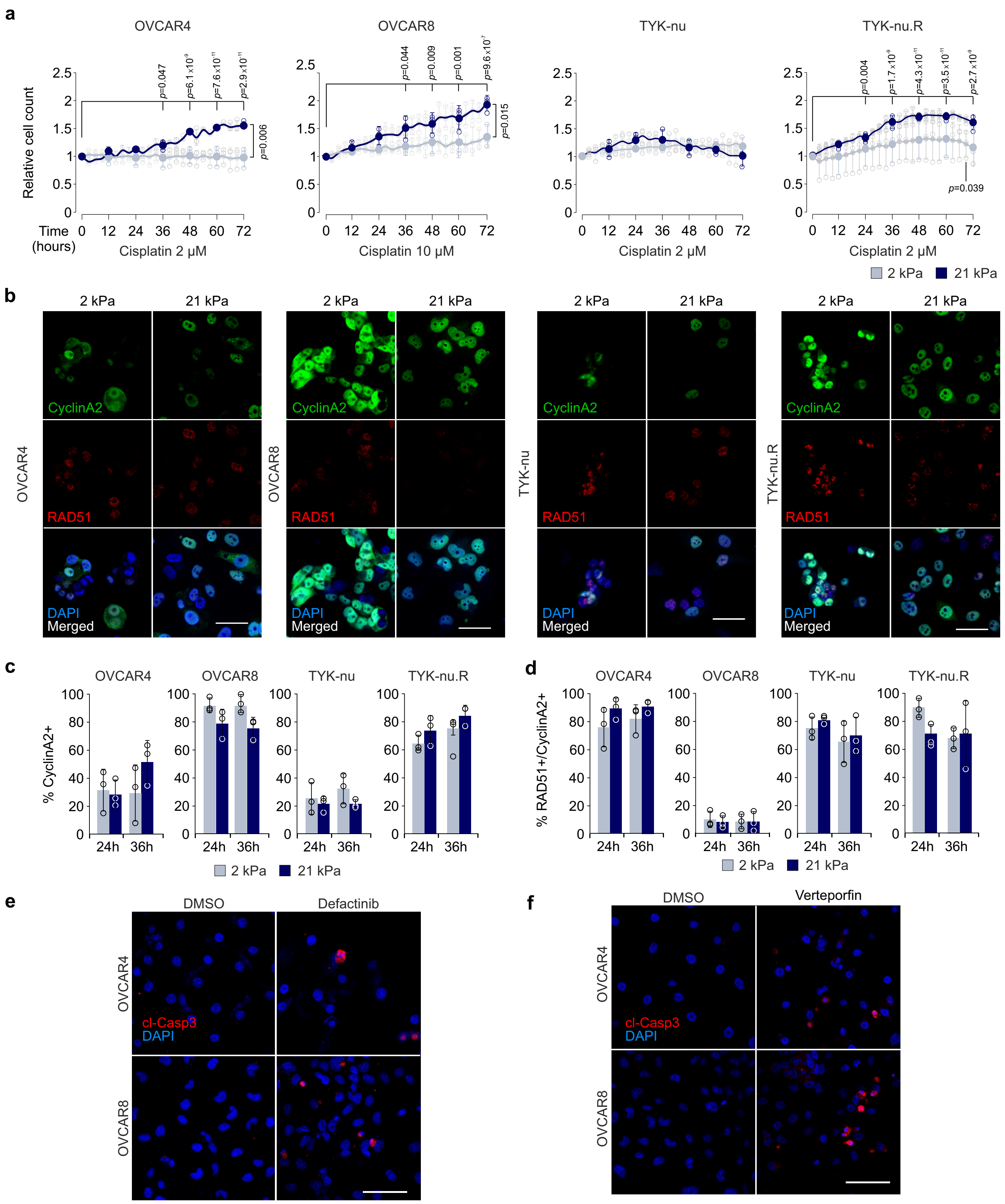

Supplementary Figure 8. DNA damage signaling accumulates in HR proficient and HR deficient cells on stiff ECM

a, Charts depict the relative cell count of corresponding cells over 72 h 2 µM (OVCAR4, TYK-nu, TYK-nu.R) or 10 µM (OVCAR8) cisplatin treatment cultured on 2 kPa and 21 kPa collagen 1 functionalized polyacrylamide (COL1-PAA) hydrogels. Cell count relative to 0 hours. See Supplementary Movies 1-4.

b, Representative confocal images of cyclinA2 (green), RAD51 (red) and co-expression (merged) in nucleus (DAPI, blue) in corresponding cells grown on 2 and 21 kPa COL1-PAA hydrogels and treated with 2 µM (OVCAR4, TYK-nu, TYK-nu.R) or 10 µM (OVCAR8) cisplatin for 24 h. Scale bar = 50 µm.

c-d, Quantification of cyclinA2+ (**c**) and RAD51+/cyclinA2+ (**d**) cells in b, and Fig. 3g.

e-f, Representative confocal micrographs for quantifications in Fig. 3h,j. Scale bar = 50 µm.

Data represent mean ± SEM; n = 3 biological replicates; two-tailed Student's t-test; one-way ANOVA with Tukey's multiple comparison test (**a**; 0h - 72h comparisons within 2 kPa and 21 kPa). Source data are provided as a Source data file.

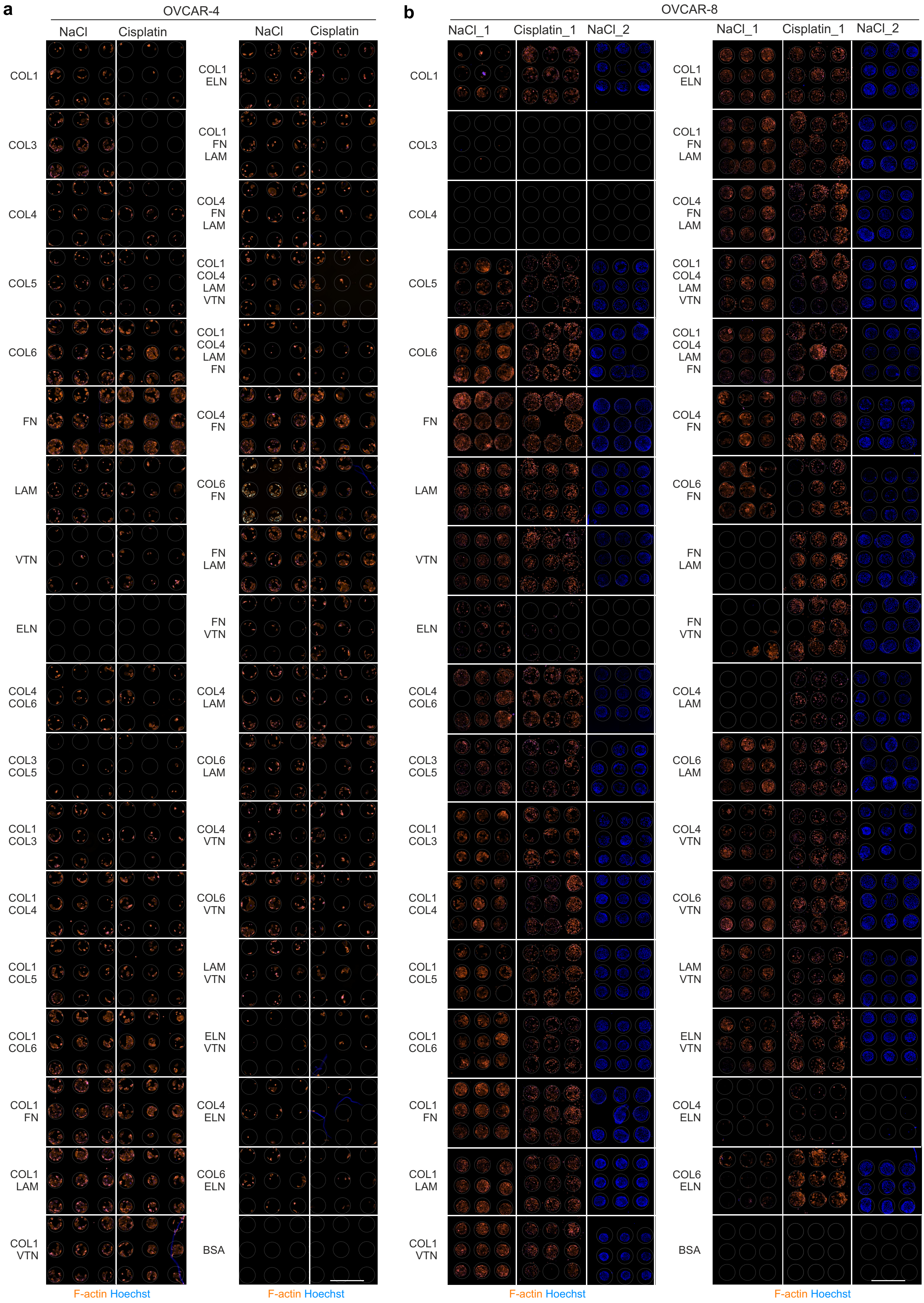
a
OVCAR-4
NaCl
Cisplatin
NaCl
Cisplatin
COL1
COL3
COL4
COL5
COL6
FN
LAM
VTN
ELN
COL4 COL6
COL3 COL5
COL1 COL3
COL1 COL4
COL1 COL5
COL1 COL6
COL1 FN
COL1 LAM
COL1 VTN
COL1 ELN
COL1 FN LAM
COL4 FN LAM
COL1 COL4 LAM VTN
COL1 COL4 LAM FN
COL4 FN
COL6 FN
FN LAM
FN VTN
COL4 LAM
COL6 LAM
COL4 VTN
COL6 VTN
LAM VTN
ELN VTN
COL4 ELN
COL6 ELN
BSA
F-actin Hoechst
F-actin Hoechst
b
OVCAR-8
NaCl_1
Cisplatin_1
NaCl_2
NaCl_1
Cisplatin_1
NaCl_2
F-actin Hoechst
F-actin Hoechst

Supplementary Figure 9. Both ECM proteins and cisplatin treatment alter HGSC cell ECM-adhesion.
a,b, Light micrographs of OVCAR4 (**a**) and OVCAR8 (**b**) grown on 2D extracellular matrix (ECM) protein array for 24 h (adherence) and treated with NaCl (control) or cisplatin (2 μM or 10 μM, respectively) for 48 h. Cells were stained for F-actin (phalloidin, orange) and nuclei (Hoechst, blue); n = 9 technical replicates; OVCAR4 n = 1 array, OVCAR8 NaCl n = 2 arrays and cisplatin n = 1 array, all with 9 technical replicates. OVCAR8 NaCl_1 and Cisplatin_1 are from the same experiment and hence comparable. COL = collagen, FN = fibronectin, LAM = laminin, VTN = vitronectin, ELN = elastin, BSA = bovine serum albumin. White outlines indicate each protein microspot. Scale bar = 200 μm.

a OVCAR4

	NaCl	Cisplatin
FN + VTN	4.5	5.5
VTN	2.7	2.9
FN	26.1	26.3
COL1 + COL3	14.5	2.3
COL1	11.0	1.3

b OVCAR8

	NaCl	Cisplatin
COL4 + ELN	1.3	4.3
COL6 + ELN	17.6	29.2
COL1	16.8	23.8
COL1 + COL4	27.5	36.5
COL1 + COL5	27.7	30.3
COL4 + VTN	28.4	30.0
ELN	14.0	4.3
COL3	0.6	0.0

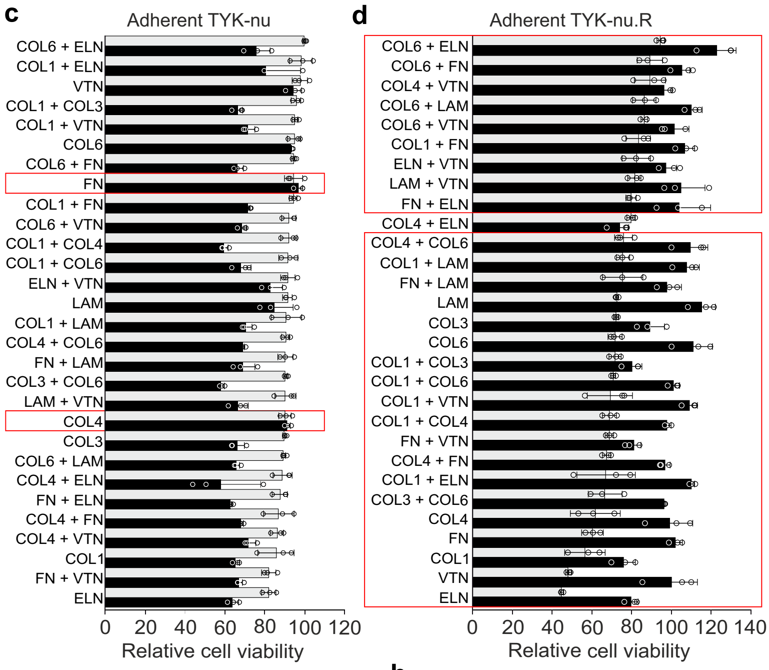

e TYK-nu

	NaCl	Cisplatin
FN	94.6	97.1
COL4	90.5	91.3
COL6	95.1	93.2
VTN	98.0	94.5
COL4 + ELN	89.2	58.0
COL1 + COL4	92.4	59.7
COL3 + COL6	90.6	58.4

f TYK-nu.R

	NaCl	Cisplatin
VTN	48.2	100.3
ELN	45.0	80.1
FN	60.5	102.5
COL4 + ELN	80.1	74.5

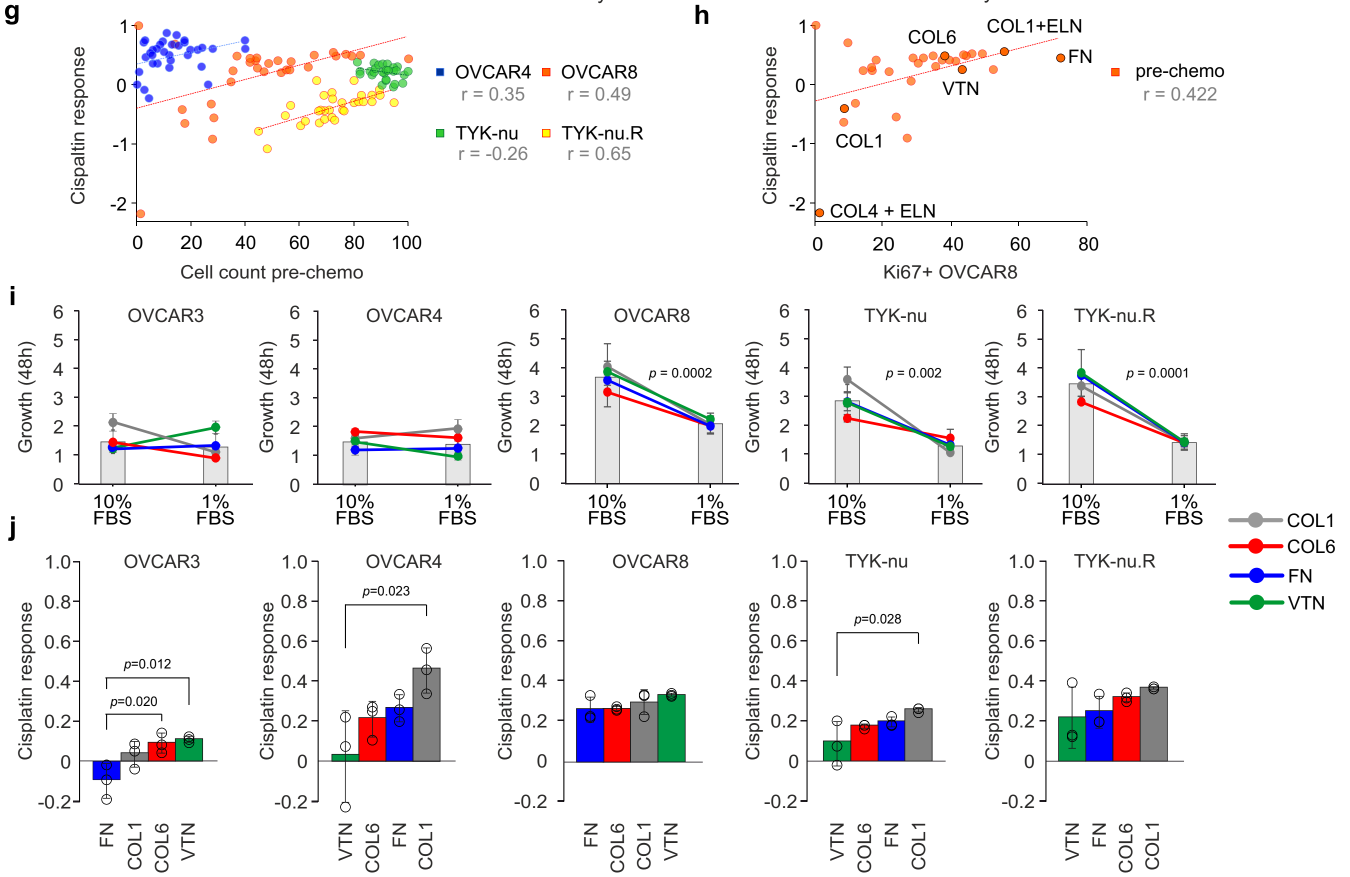

Supplementary Figure 10. ECM proteins affect cisplatin response of HGSC cells.
a,b, Tables show relative cell count of OVCAR4 (**a**) and OVCAR8 (**b**) grown on 2D extracellular matrix (ECM) protein array for 24 h (adherence) and treated with NaCl (control) or cisplatin (2 µM or 10 µM, respectively) for 48 h. Red indicates negative cisplatin response (higher cell count with cisplatin treatment than with NaCl) and blue indicates positive response; n = 1 array with 9 technical replicates. See Supplementary Fig. 9 for ECM array light micrographs and Fig. 4a,b, for full quantification. COL = collagen, FN = fibronectin, VTN = vitronectin, ELN = elastin.
c,d, Charts illustrate relative viability of TYK-nu (**c**) and TYK-nu.R (**d**) grown on 29 different single ECM protein or combination substrates for 24 h (adherence) and treated with NaCl (control) or 2 µM cisplatin for 48 h. Cell viability was determined by ATP measurement. Data is presented relative to untreated TYK-nu cell viability in COL6 + ELN. Data represent mean ± SEM; n = 3 biological replicates. Red boxes indicate the conditions that result in higher cell viability after cisplatin treatment compared to NaCl. LAM = laminin.
e,f, Tables show relative cell viability of TYK-nu (**e**) and TYK-nu.R (**f**) in c and d. Red indicates negative cisplatin response (higher cell count with cisplatin treatment than with NaCl) and blue indicates positive response.
g,h, Scatter plots depict the correlation of cisplatin response against pre-chemo cell count (OVCAR4, blue, and OVCAR8, orange; n = 1 array with 9 technical replicates) and cell viability (TYK-nu, green, and TYK-nu.R, yellow; n = 3 biological replicates) (**g**) and against ki67 positivity in OVCAR8 pre-chemotherapy (**h**; n = 1 array, with 9 technical replicates); two-tailed Pearson correlation.
I, Charts illustrate the change in 48 h growth of corresponding cells on 2D COL1 (grey), COL6 (red), FN (blue) and VTN (green) with 10% and 1 % fetal bovine serum (FBS). Growth (48 h) was calculated by [cell count (72 h) - cell count (24 h)] / cell count (24 h). Data represent mean ± SEM; n = 3 technical replicates; two-tailed Student's t-test.
j, Charts illustrate cisplatin response of corresponding cells on COL1 (grey), COL6 (red), FN (blue) and VTN (green) with 1 % FBS. Cisplatin response was calculated by [cell count (NaCl) - cell count (cisplatin)] / cell count (NaCl). Data represent mean ± SEM; n = 3 biological replicates; one-way ANOVA with Tukey's multiple comparison test.
Source data are provided as a Source data file.

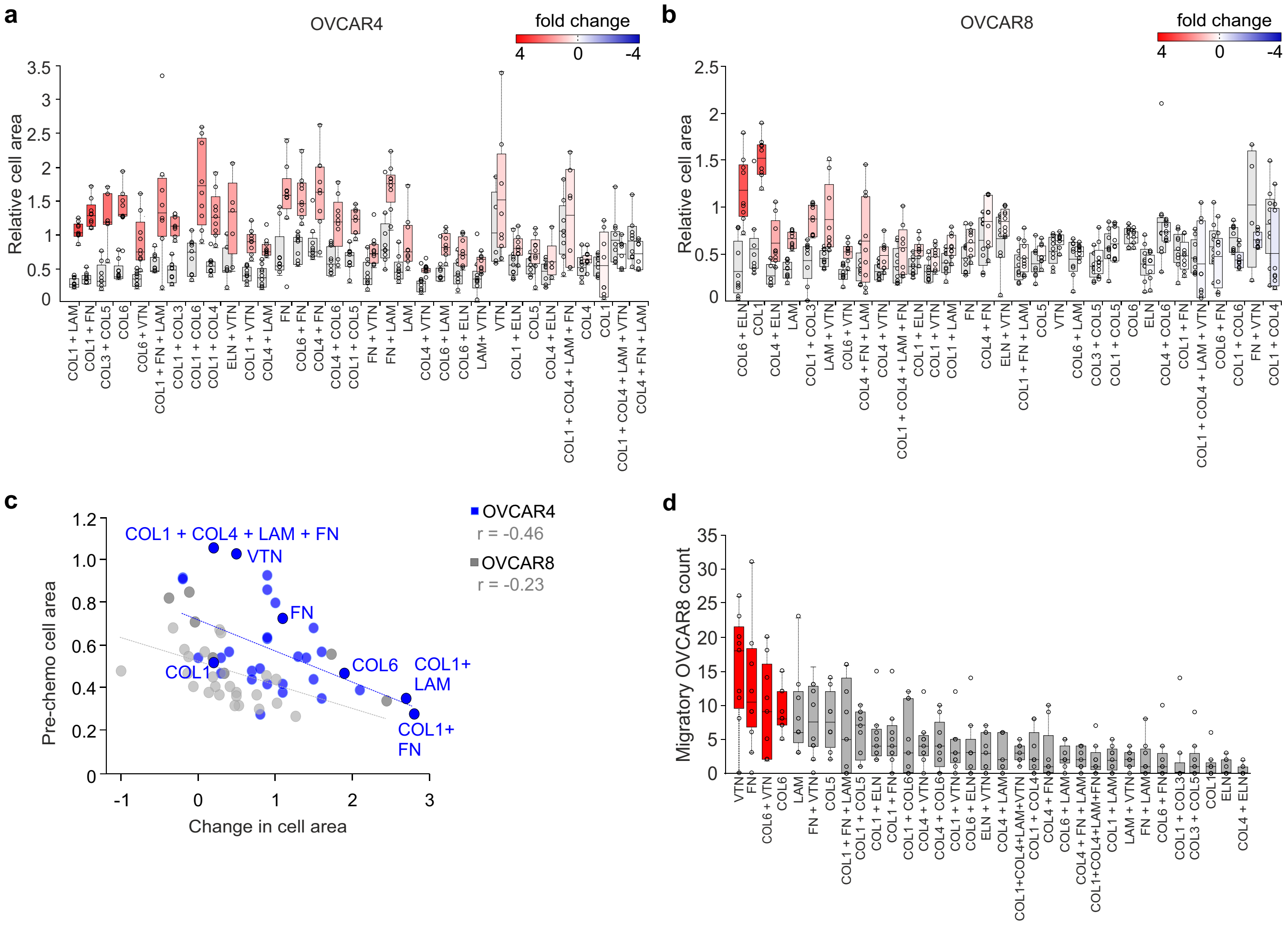

Supplementary Figure 11. Cisplatin treatment and ECM proteins affect HGSC cytoskeletal features.

a,b, Charts illustrate relative area of OVCAR4 (**a**) and OVCAR8 (**b**) grown on 2D extracellular matrix (ECM) protein array for 24 h (adherence) and treated with NaCl (control; grey boxes) or cisplatin (2 µM or 10 µM, respectively; colored boxes) for 48 h. Color scale illustrates the fold change in cell area between NaCl and cisplatin treatment. COL = collagen, FN = fibronectin, LAM = laminin, VTN = vitronectin, ELN = elastin.

c, Scatter plot shows the correlation of relative cell area pre-chemotherapy against the change in cell area induced by cisplatin treatment in OVCAR4 (blue) and OVCAR8 (grey); two-tailed Pearson correlation.

d, Chart illustrates migratory OVCAR8 count on ECM protein array upon 48 h 10 µM cisplatin treatment. Red-colored plots indicate the four most migration-inducing ECM protein substrates.

Box plots indicate median (middle line), 25th, 75th percentile (box) and 10th and 90th percentile (whiskers) as well as outliers (single points); n = 1 array with 9 technical replicates. Source data are provided as a Source data file.

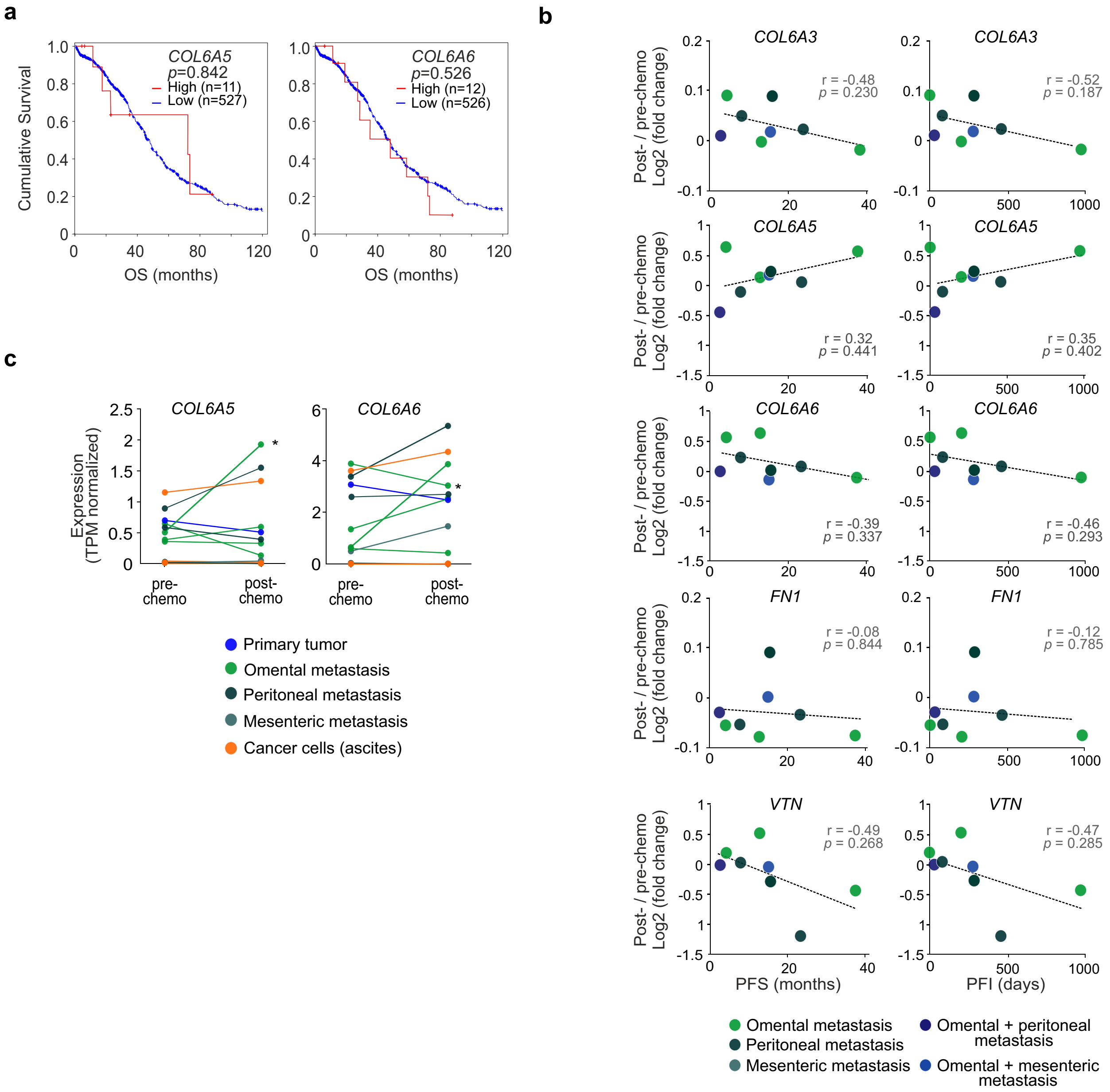

Supplementary Figure 12. Collagen 6 is mostly expressed by stromal cells.
a, Kaplan-Meier curves illustrate the association between collagen 6A5 (*COL6A5*) and *COL6A6* expression in chemo-naïve ovarian cancer tissues with overall survival (OS) in The Cancer Genome Atlas (TCGA) dataset; log-rank test.
b, Scatter plots depict the correlation between *COL6A3-A6*, fibronectin (*FN1*) and vitronectin (*VTN*) expression fold change (post-chemotherapy against pre-chemotherapy) with platinum-free interval (PFI) and progression free survival (PFS) in matched patient and matched tissue samples (n = 8); two-tailed Pearson correlation.
c, Chart illustrates change in *COL6A5* and *COL6A6* upon chemotherapy in matched high-grade serous carcinoma (HGSC) patient-derived samples (n = 12) from initially platinum-sensitive patients (including partial / complete response or stable disease). Asterisk identifies patient with increased platinum-free interval (974 days) in comparison to other patients (0-460 days). No significant difference between the expression in pre- and post-chemotherapy metastatic tissues; two-tailed Student's t-test. See Supplementary Data 29 for exact values.

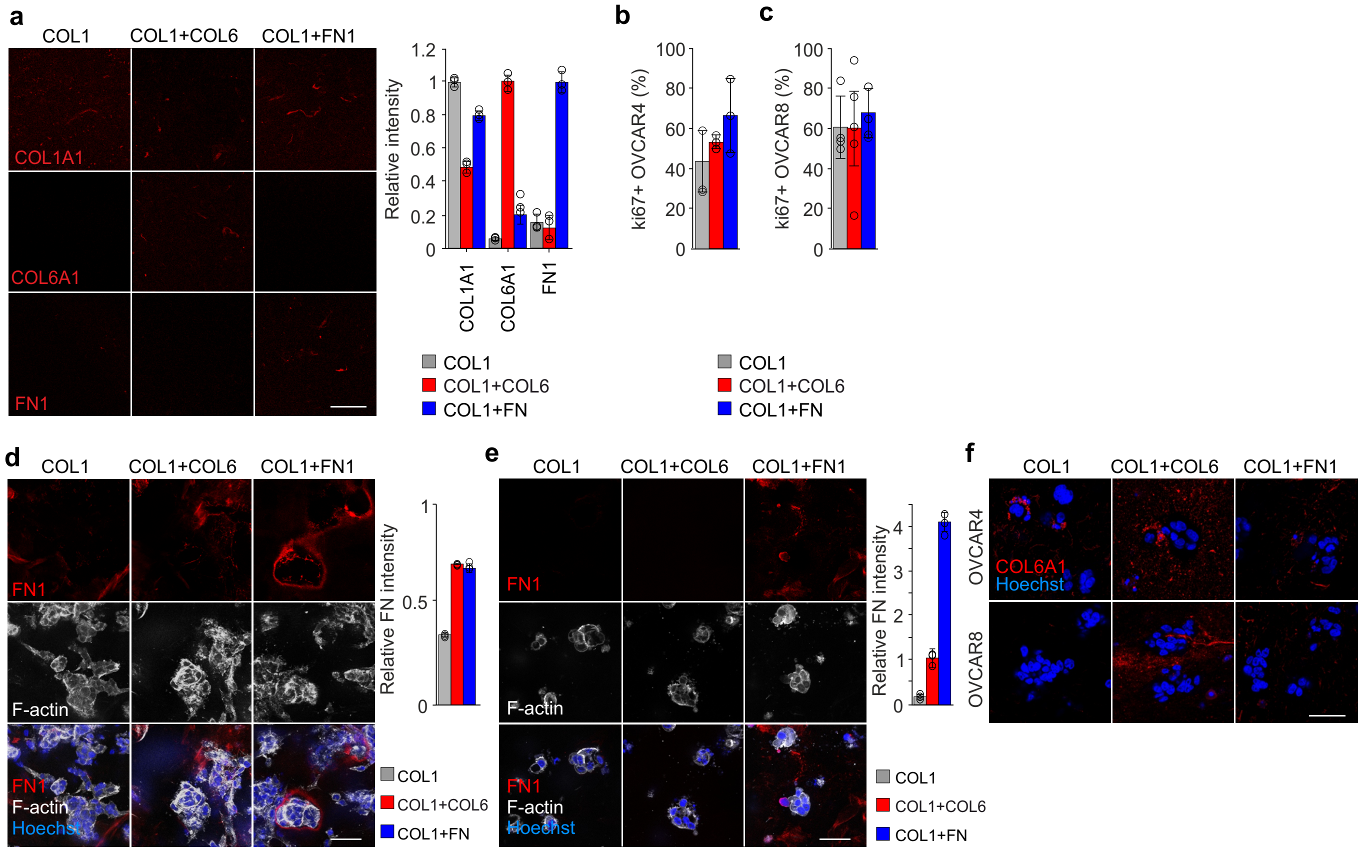

Supplementary Figure 13. More mesenchymal OVCAR8 cells deposit or retain fibronectin in the ECM.
a, Representative confocal micrographs and quantifications of collagen 1A1 (COL1A1, red), COL6A1 (red) and fibronectin (FN1, red) in 3D COL1 matrix, and in COL1 matrices supplemented with 50 µg/ml COL6 or FN. Scale bar = 100 µm.
b,c, Quantification of Ki67 positivity in OVCAR4 (**b**) and OVCAR8 (**c**) grown in corresponding 3D matrices for 5 d, as shown in micrographs in Fig. 6c.
d,e, Representative confocal micrographs and quantifications of FN1 (red) and F-actin (phalloidin, white) in corresponding 3D cultures of OVCAR4 (**d**) and OVCAR8 (**e**) after 5 d. Scale bar = 50 µm.
f, Representative confocal micrographs of COL6A1 in corresponding 3D cultures of OVCAR4 and OVCAR8 after 5 d. Scale bar = 50 µm.
Data represent mean ± SEM; n = 3 (**a, b, d-f**) and n = 4 (**c**) biological replicates; two-tailed Student's t-test. Source data are provided as a Source Data file.

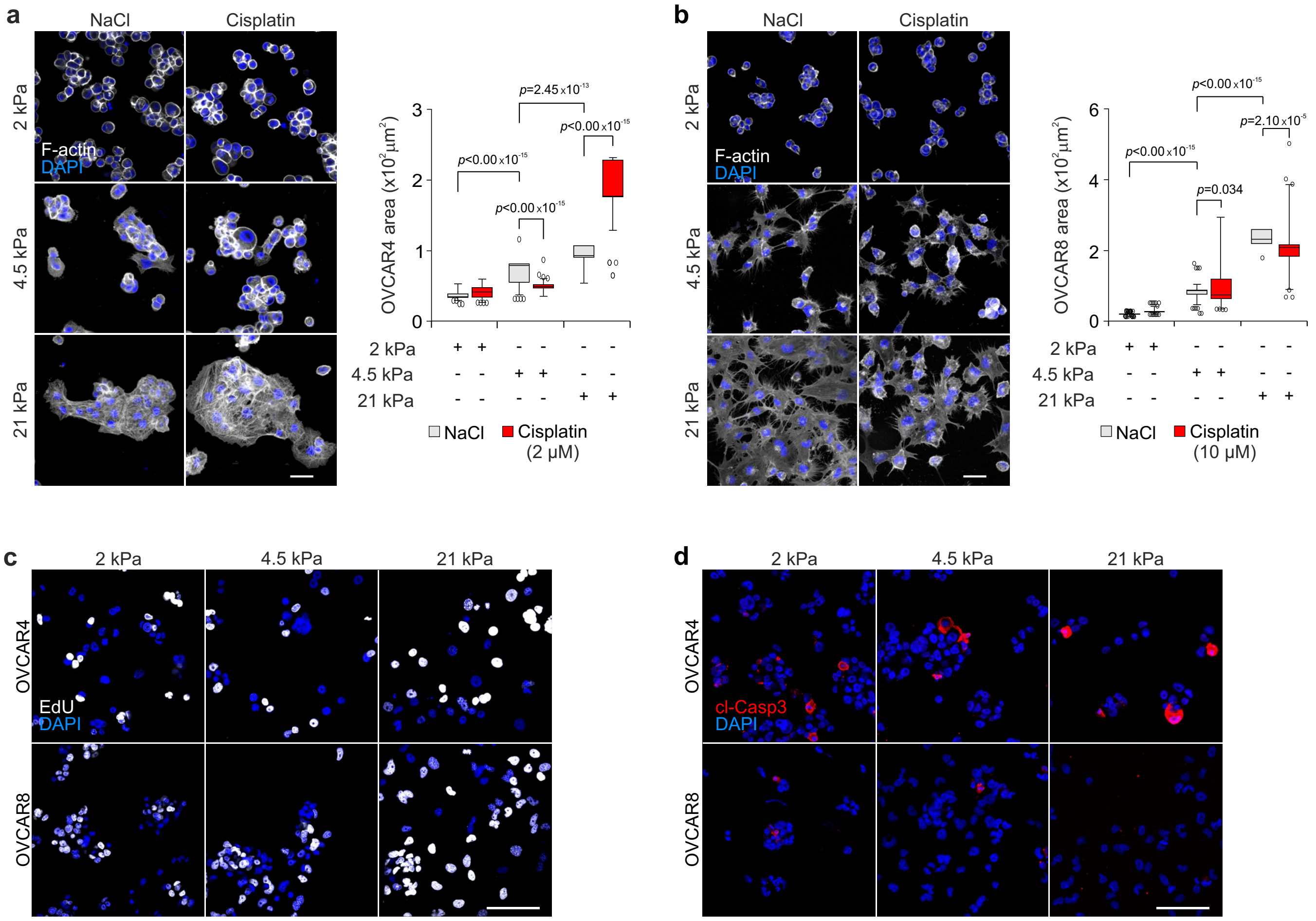

Supplementary Figure 14. Collagen 6 protects intrinsically resistant cells against cisplatin-induced apoptosis.
a,b, Representative confocal micrographs of F-actin (phalloidin, white) and corresponding quantification of OVCAR4 (**a**) and OVCAR8 (**b**) area on 2, 4.5 and 21 kPa collagen 6-functionalized polyacrylamide hydrogels (COL6-PAA) at 48 h; OVCAR4 n = 108/143, 108/119 and 58/69, and OVCAR8 n=177/144, 163/99 and 85/59 cells, respectively. Data represent mean ± SEM of 3 biological replicates; one-way ANOVA with Tukey's multiple comparison test. Box plots indicate median (middle line), 25th, 75th percentile (box) and 10th and 90th percentile (whiskers) as well as outliers (single points). Scale bar = 25 µm.
c,d, Representative confocal micrographs of EdU (**c**; white, marker of proliferation) and cleaved caspase 3 (cl-Casp3, **d**; red; marker of apoptosis) in OVCAR4 and OVCAR8 grown on 2 kPa, 4.5 kPa and 21 kPa COL6-PAA; n = 3 biological replicates. For quantifications see Fig. 7e,f.
Source data are provided as a Source Data file.

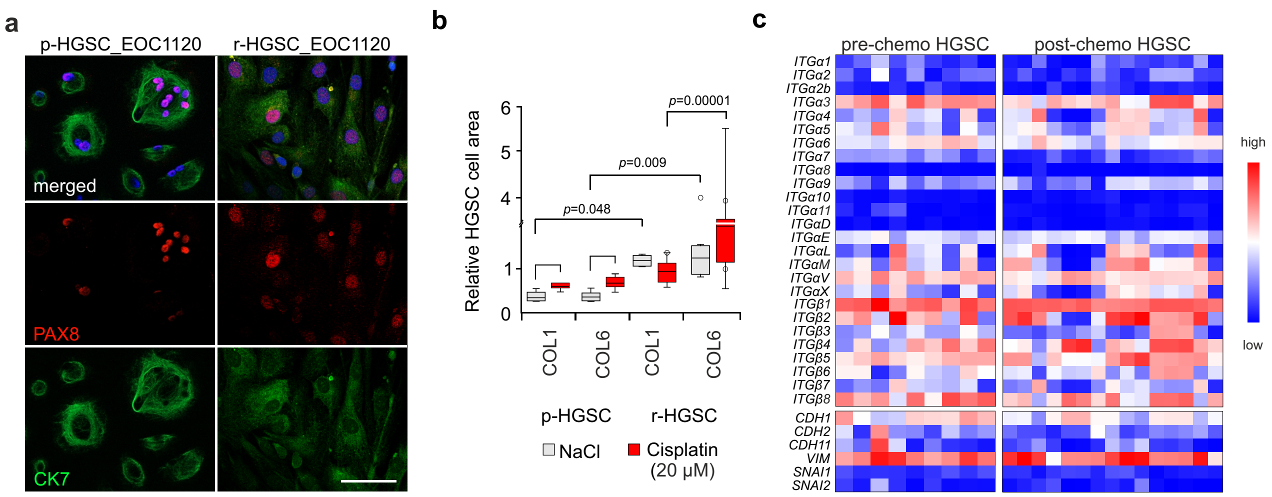

Supplementary Figure 15. Collagen 6 confers relapse HGSC ascites-derived cells with cisplatin-induced adhesion and resistance.
a, Representative confocal micrographs of PAX8 (red) and cytokeratin 7 (CK7, green) in EOC1120 patient ascites-derived cells pre-chemotherapy (p-HGSC) and at relapse (r-HGSC). Scale bar = 50 µm.
b, Quantification of p-HGSC and r-HGSC relative cell spreading area. Box plots indicate median (middle line), 25th, 75th percentile (box) and 10th and 90th percentile (whiskers) as well as outliers (single points); n = 6 distinct patient samples from 5 patients (3 x pre-chemo EOC1032, EOC691 and EOC1120 p_HGSC; 3 x post-chemo EOC1120, EOC167 and EOC26 r-HGSC) with n = 3 biological replicates per patient combined with mean value for analysis; one-way ANOVA with Tukey's multiple comparison test.
c, Heat map illustrates integrin (ITG; low = 0.00, high = 10.20) and epithelial-mesenchymal marker (low = 0.00, high = 11.40) expression in HGSC ascites-derived cells determined by RNAseq; n = 9 pre-chemotherapy HGSC patient samples, n = 15 post-chemotherapy HGSC patient samples. Color key identifies the normalized gene expression values.
Source data are provided as a Source Data file.
