# Supplementary material for: Co-evolution of matrisome and adaptive adhesion dynamics drives ovarian cancer chemoresistance
Source: Nat Commun. 2021 Jun 23;12:3904. doi: 10.1038/s41467-021-24009-8 (PMC8222388; doi:10.1038/s41467-021-24009-8)
Supplement: Supplementary file 3 — Description of Additional Supplementary Files [file 41467_2021_24009_MOESM3_ESM.pdf]

**Description of Additional Supplementary Files**

File Name: Supplementary Data 1
Description: Patient information.

File Name: Supplementary Data 2
Description: Summary of patient information.

File Name: Supplementary Data 3
Description: Shared DEGs encoding matrisome proteins in pre-chemotherapy HGSC metastatic tissues against primary tumor tissue.

File Name: Supplementary Data 4
Description: DEGs encoding matrisome proteins in pre-chemotherapy HGSC patient-derived omental metastasis against primary tumor tissue.

File Name: Supplementary Data 5
Description: DEGs encoding matrisome proteins in pre-chemotherapy HGSC patient-derived peritoneal metastasis against primary tumor tissue.

File Name: Supplementary Data 6
Description: DEGs encoding matrisome proteins in pre-chemotherapy HGSC patient-derived mesenteric metastasis against primary tumor tissue.

File Name: Supplementary Data 7
Description: DEGs encoding matrisome proteins in pre-chemotherapy HGSC patient-derived solid tumors against ascites-derived cells.

File Name: Supplementary Data 8
Description: DEGs encoding matrisome proteins in HGSC patient-derived pre-chemotherapy omental and peritoneal metastases against pre-chemotherapy primary tumor tissue.

File Name: Supplementary Data 9
Description: Enriched canonical pathways identified in pre-chemotherapy omental and peritoneal metastasis against primary tumor.

File Name: Supplementary Data 10
Description: Shared DEGs encoding matrisome proteins in post-chemotherapy HGSC tissues against their pre-chemotherapy counterpart.

File Name: Supplementary Data 11
Description: DEGs encoding matrisome proteins in HGSC patient-derived post-chemotherapy primary tumor against pre-chemotherapy primary tumor tissue.

File Name: Supplementary Data 12
Description: DEGs encoding matrisome proteins in HGSC patient-derived post-chemotherapy omental metastases against pre-chemotherapy omental metastatic tissue.

File Name: Supplementary Data 13
Description: DEGs encoding matrisome proteins in HGSC patient-derived post-chemotherapy peritoneal metastases against pre-chemotherapy peritoneal metastatic tissue.

File Name: Supplementary Data 14
Description: DEGs encoding matrisome proteins in HGSC patient-derived post-chemotherapy mesenteric metastases against pre-chemotherapy mesenteric metastatic tissue.

File Name: Supplementary Data 15
Description: DEGs encoding matrisome proteins in HGSC patient-derived post-chemotherapy omental and peritoneal metastases against pre-chemotherapy omental and peritoneal metastases.

File Name: Supplementary Data 16
Description: DEGs encoding matrisome proteins in HGSC patient-derived post-chemotherapy ascites-derived cells against pre-chemotherapy ascites-derived cells.

File Name: Supplementary Data 17
Description: DEGs encoding matrisome proteins in HGSC patient-derived post-chemotherapy omental and peritoneal metastases against post-chemotherapy primary tumor tissue.

File Name: Supplementary Data 18
Description: Enriched canonical pathways identified in post-chemotherapy omental and peritoneal metastasis against post-chemotherapy primary tumor.

File Name: Supplementary Data 19
Description: DEGs encoding matrisome proteins in pre-chemotherapy primary tissues from HGSC patients with platinum free interval (PFI) time less than 6 months against PFI more than 6 months.

File Name: Supplementary Data 20
Description: DEGs encoding matrisome proteins in post-chemotherapy primary tissues from HGSC patients with platinum free interval (PFI) time less than 6 months against PFI more than 6 months.

File Name: Supplementary Data 21
Description: DEGs encoding matrisome proteins in pre-chemotherapy metastatic tissues from HGSC patients with platinum free interval (PFI) time less than 6 months against PFI more than 6 months.

File Name: Supplementary Data 22

Description: DEGs encoding matrisome proteins in post-chemotherapy metastatic tissues from HGSC patients with platinum free interval (PFI) time less than 6 months against PFI more than 6 months.

File Name: Supplementary Data 23
Description: Shared DEGs encoding matrisome proteins in HGSC primary tumor and metastatic tissues from patients with platinum-free interval (PFI) less than 6 months against PFI more than 6 months.

File Name: Supplementary Data 24
Description: DEGs encoding matrisome proteins in pre-chemotherapy ascites-derived cancer cells from HGSC patients with platinum free interval (PFI) time less than 6 months against PFI more than 6 months.

File Name: Supplementary Data 25
Description: DEGs encoding matrisome proteins in post-chemotherapy ascites-derived cancer cells from HGSC patients with platinum free interval (PFI) time less than 6 months against PFI more than 6 months.

File Name: Supplementary Data 26
Description: Gene expression of COL6A1, COL6A2, COL6A3, COL6A5, COL6A6, FN1 and VTN in HGSC patient-derived omental, peritoneal and mesenteric metastatic tissues and in primary tumor tissue.

File Name: Supplementary Data 27
Description: DEGs encoding matrisome proteins in HGSC patient-derived pre-chemotherapy omental+peritoneal+mesenteric metastatic tissues against primary tumor tissue.

File Name: Supplementary Data 28
Description: DEGs encoding matrisome proteins in HGSC patient-derived post-chemotherapy omental+peritoneal+mesenteric metastatic tissues against primary tumor tissue.

File Name: Supplementary Data 29
Description: Fold change (post-chemotherapy to pre-chemotherapy) of transcript-per-million normalized gene expression of COL1, COL6, FN and VTN against progression-free survival (PFS) and platinum-free interval (PFI) in matching HGSC samples.

File Name: Supplementary Movie 1
Description: Time-lapse video of cisplatin treated OVCAR4 on soft and stiff hydrogels. The cells were labelled for cleaved caspase 3/7 (Cl-casp; green). Cell movement was recorded by time-lapse microscopy using Cytation 5 microscope (Biotek). Frames taken every 3 h for 72 h are displayed at 1 frame-per-second.

File Name: Supplementary Movie 2
Description: Time-lapse video of cisplatin treated OVCAR8 on soft and stiff hydrogels. The cells were labelled for cleaved caspase 3/7 (Cl-casp 3/7; green). Cell movement was recorded by time-lapse microscopy using Cytation 5 microscope (Biotek). Frames taken every 3 h for 72 h are displayed at 1 frame-per-second.

File Name: Supplementary Movie 3
Description: Time-lapse video of cisplatin treated TYK-nu on soft and stiff hydrogels. The cells were labelled for cleaved caspase 3/7 (Cl-casp 3/7; green). Cell movement was recorded time-lapse microscopy using Cytation 5 microscope (Biotek). Frames taken every 3 h for 72 h are displayed at 1 frame-per-second.

File Name: Supplementary Movie 4
Description: Time-lapse video of cisplatin treated TYK-nu.R on soft and stiff hydrogels. The cells were labelled for cleaved caspase 3/7 (Cl-casp 3/7; green). Cell movement was recorded time-lapse microscopy using Cytation 5 microscope (Biotek). Frames taken every 3 h for 72 h are displayed at 1 frame-per-second.
