# Supplementary material for: Co-evolution of matrisome and adaptive adhesion dynamics drives ovarian cancer chemoresistance
Source: Nat Commun. 2021 Jun 23;12:3904. doi: 10.1038/s41467-021-24009-8 (PMC8222388; doi:10.1038/s41467-021-24009-8)
Supplement: Supplementary file 9 — Reporting Summary [file 41467_2021_24009_MOESM9_ESM.pdf]

nature research

Corresponding author(s): Kaisa Lehti

Last updated by author(s): May 17, 2021

Reporting Summary

Nature Research wishes to improve the reproducibility of the work that we publish. This form provides structure for consistency and transparency in reporting. For further information on Nature Research policies, see our Editorial Policies and the Editorial Policy Checklist.

Statistics

For all statistical analyses, confirm that the following items are present in the figure legend, table legend, main text, or Methods section.

n/a	Confirmed	
☐	☒	The exact sample size (n) for each experimental group/condition, given as a discrete number and unit of measurement
☐	☒	A statement on whether measurements were taken from distinct samples or whether the same sample was measured repeatedly
☐	☒	The statistical test(s) used AND whether they are one- or two-sided<br>*Only common tests should be described solely by name; describe more complex techniques in the Methods section.*
☐	☒	A description of all covariates tested
☐	☒	A description of any assumptions or corrections, such as tests of normality and adjustment for multiple comparisons
☐	☒	A full description of the statistical parameters including central tendency (e.g. means) or other basic estimates (e.g. regression coefficient) AND variation (e.g. standard deviation) or associated estimates of uncertainty (e.g. confidence intervals)
☐	☒	For null hypothesis testing, the test statistic (e.g. F, t, r) with confidence intervals, effect sizes, degrees of freedom and P value noted<br>*Give P values as exact values whenever suitable.*
☒	☐	For Bayesian analysis, information on the choice of priors and Markov chain Monte Carlo settings
☒	☐	For hierarchical and complex designs, identification of the appropriate level for tests and full reporting of outcomes
☐	☒	Estimates of effect sizes (e.g. Cohen's d, Pearson's r), indicating how they were calculated

Our web collection on statistics for biologists contains articles on many of the points above.

Software and code

Policy information about availability of computer code

Data collection	Micrographs were obtained using ZEN 2.3 and 2.6 Blue as well as ZEN 2.3 Black edition software (Zeiss). Live cell imaging was done by using Cytation 5 imaging reader (BioTek, CYT5MPV). Immunohistochemistry images were obtained using Pannoramic Viewer 2.0 - 250 (3DHISTECH Ltd.). Immunoblots were obtained using Image Studio Lite Ver 5.2 (LI-COR Biosciences)
Data analysis	Fji-ImageJ 1.52p (FibrilToll plug.in), QuPath v0.1.2 and CellProlifer 3.1.8 were used for image-based quantifications. Statistical analysis were perfomed using IBM SPSS Statistics 25 and Graphpad Prism 7. For RNAseq analysis the following softwares were used: Trimmomatic V0.33, STAR V2.5.2b, eXpress V1.5.1-linux_x86_64, DESeq2 V1.22.1 and IPA (QIAGEN Inc.).

For manuscripts utilizing custom algorithms or software that are central to the research but not yet described in published literature, software must be made available to editors and reviewers. We strongly encourage code deposition in a community repository (e.g. GitHub). See the Nature Research guidelines for submitting code & software for further information.

Data

Policy information about availability of data

All manuscripts must include a data availability statement. This statement should provide the following information, where applicable:

- Accession codes, unique identifiers, or web links for publicly available datasets
- A list of figures that have associated raw data
- A description of any restrictions on data availability

All processed RNA sequencing data used for the analysis in this study (Figures 1b-1e, 2a-e, 5b-d and Supplementary Figures 1a-b, 2, 3b-e, 12 b-c, 15c) is available in public Gene Expression Omnibus (GEO) database under the accession code GSE173420 [https://www.ncbi.nlm.nih.gov/geo/query/acc.cgi?acc=GSE173420]. The corresponding raw RNA sequencing data is available in the European Genome-Phenome Archive (EGA) with the accession code EGAD00001006456 [https://ega-archive.org/datasets/EGAD00001006456], under the study EGAS00001004714 [https://ega-archive.org/studies/EGAS00001004714]. Restrictions due to EU General

Data Protection Regulation (GDPR) forbid to grant access to anonymous persons. Thus, the raw data is available upon request from the Data Access Committee at sysbio-dac@helsinki.fi. The dataset of 32 epithelial HGSC and 31 stromal samples are publicly available at GEO under accession code GSE40595 [https://www.ncbi.nlm.nih.gov/geo/query/acc.cgi?acc=GSE40595] (Figure 5e). The Cancer Genome Atlas ovarian serous cystadenocarcinoma patient dataset (TCGA, Firehose Legacy) is available at [https://portal.gdc.cancer.gov/projects/TCGA-OV] (Figures 5a and Supplementary Figure12a). The remaining data are available within the Article and Supplementary Information. Source Data are provided with this paper.

Field-specific reporting

Please select the one below that is the best fit for your research. If you are not sure, read the appropriate sections before making your selection.

- [x] Life sciences
- [ ] Behavioural & social sciences
- [ ] Ecological, evolutionary & environmental sciences

For a reference copy of the document with all sections, see nature.com/documents/nr-reporting-summary-flat.pdf

Life sciences study design

All studies must disclose on these points even when the disclosure is negative.

Sample size	No statistical methods were used to pre-determine sample size. Minimum number of technical and biological replicates was set to 3 based on researchers experience. For transcriptomic analyses, all available high-grade serous ovarian carcinoma samples were used. For ex vivo experiments all viable pre- and post-chemotherapy patient ascites-derived cells. were used. For in vitro experiments the n numbers were determined according to the minimal number of independent biological replicates that significantly identify an effect.
Data exclusions	From differential expressed gene analysis of genes encoding matrisome proteins (Fig. 1,2; Supplementary Fig.1,2,3), total of two genes (CCL4L1 and MUC8) were excluded due to missing gene identifier. From platinum-free interval and progression-free survival analysis (Fig. 5 and Supplementary Fig. 12) total of one patient was excluded due to death from non-cancer related reasons during chemotherapy treatment.
Replication	All experiments in this study were independently replicated, with technical and biological replicates if possible, achieving similar results. The number and type of replicate is listed in the legends of the corresponding figures.
Randomization	No preallocation consideration was done for the in vitro and ex vivo experiments; samples were randomly subjected to treatment or control.
Blinding	As potential sources of biasness were not identifiable investigators were not blinded to allocation during experiments and outcome assessment. Also, blinding was not possible as the same investigator processed the samples and analysed the data.

Reporting for specific materials, systems and methods

We require information from authors about some types of materials, experimental systems and methods used in many studies. Here, indicate whether each material, system or method listed is relevant to your study. If you are not sure if a list item applies to your research, read the appropriate section before selecting a response.

Materials & experimental systems

n/a	Involved in the study	
[ ]	[x]	Antibodies
[ ]	[x]	Eukaryotic cell lines
[x]	[ ]	Palaeontology and archaeology
[x]	[ ]	Animals and other organisms
[ ]	[x]	Human research participants
[x]	[ ]	Clinical data
[x]	[ ]	Dual use research of concern

Methods

n/a	Involved in the study	
[x]	[ ]	ChIP-seq
[x]	[ ]	Flow cytometry
[x]	[ ]	MRI-based neuroimaging

Antibodies

Antibodies used	Cytokeratin 7 (ImmunoWay, YM3054, Lot. B5401), Collagen type I alpha 1 (Abcam, ab34710, Lot.GR287695-1), Collagen type VI alpha 1 (Abcam, ab6588, Lot.GR203100-10; clone B-4, Santa Cruz, sc-377143, Lot. K0716), Fibronectin (Sigma-Aldrich, F3648-100UL, Lot. 125M4835V), Phosphorylated (S139) gamma H2AX (Cell Signaling Technologies, #2577S, Lot. 11; Abcam, ab22551, Lot. GR3373372-1), Active-integrin β1 (Abcam, ab30394, 2G10, GR305468-1), PAX8 (Proteintech, 10336-1-AP, Lot. 00050931), Ki67 (Leica Biosystems, NCL-Ki67p, Lot. 6013874), Cleaved caspase-3 (Asp175) (Cell Signaling Technologies, #9664S, 5A1E, Lot. 22), Phosphorylated (Y397) FAK (BD Biosciences, 611807, Lot. 6092601), Phosphorylated (S20) myosin light chain (Abcam, ab2480, Lot. GR3241064-7), YAP/TAZ (Santa Cruz Biotechnology, sc-101199, 63.7, Lot. J3013), CD68 (Sigma-Aldrich, HPA_048982, Lot. 000002294), CD45 (clone 2B11 + PD7/26, Dako, M070129-2, Lot. 20026786), FSP1 (S100A4, Proteintech, 16105-1-AP), β-actin (clone C-4, Santa Cruz, sc-47778, Lot. K1617), RAD51 (Abcam, ab133534, Lot. GR3270300-7), CyclinA2 (Gene-Tex, GTX6234420, Lot. 43024), Caspase-3/7 (Invitrogen, C10723, Lot. 2208514).
Validation	All antibodies used in this study are commercially available and have been validated for the application by the manufacturer or by specific references found on the suppliers homepage.

Eukaryotic cell lines

Policy information about cell lines

Cell line source(s)	OVCAR3, OVCAR4 and OVCAR8: National Cancer Institute, U.S. TYK-nu and TYK-nu.R: Japanese Collection of Research Bioresources Cell Bank, Osaka, Japan. CCL-137 and WI-38: American Type Culture Collection, U.S.
Authentication	None of the cell lines were authenticated.
Mycoplasma contamination	The cell lines used in this study were routinely tested negative for mycoplasm.
Commonly misidentified lines (See ICLAC register)	None of the used cell lines are listed in the ICLAC database of commonly misidentified cell lines.

Human research participants

Policy information about studies involving human research participants

Population characteristics	This study includes data of a total of 71 patients. Of these patients 67 female were diagnosed with high-grade serous ovarian carcinoma, 1 with clear cell carcinoma, 2 female and 1 male with non-gynaecological cancers (referred as controls). Mean age of the patients with gynaecological cancer was 67.8 $\pm$ 4.0 . Treatment categories and further clinical information can be found in Supplementary Data 1.
Recruitment	All EOC-identified patients were recruited at the Turku University Hospital, Finland, by the medical coauthors. Patients with suspected ovarian malignancies were recruited and all eligible patients were included into the study, unless patient did not give informed consent. The histopathological diagnosis and disease stage were affirmed from tissue samples obtained during diagnostic surgery by a pathologist specialized in gynaecological pathology with the guideline of The International Federation of Gynecologists and Obstetricians (FIGO 2014). All samples were taken during necessary surgical interventions.<br>For eliminating biasness, all samples were handled with coded ID-numbers given immediately after operation. Ex vivo experiments were assessed and evaluated without the knowledge of the long-term clinical chemotherapy outcome and patient survival. All experiments were assessed within the same time limit after receiving the sample in order to eliminate time-biasness.<br>OCKI-identified patients were recruited at the Karolinska University Hospital, Sweden, as described by Moyano-Galceran et al in "Adaptive RSK-EphA2-GPRC5A signaling switch triggers chemotherapy resistance in ovarian cancer", EMBO Mol Med (DOI: 10.15252/emmm.201911177).
Ethics oversight	All studies involving clinical material were performed in accordance with the ethical standards from the 1975 Declaration of Helsinki. Each patient gave written informed consent and the use of clinical material was approved by the Ethics Committee of the Hospital District of Southwest Finland (ETMK), The National Supervisory Authority for Welfare and Health (Valvira), The Swedish Ethical Review Agency (Etikprövningsmyndigheten) and Auria Biobank (for the use of control omentum tissues).

Note that full information on the approval of the study protocol must also be provided in the manuscript.
